# Identification and characterization of immunoglobulin tau (IgT) in Asian Seabass (*Lates calcarifer)* and mucosal immune response to nervous necrosis virus

**DOI:** 10.3389/fimmu.2023.1146387

**Published:** 2023-02-20

**Authors:** Janlin Chan, Lee Ching Pei Carmen, Si Qi Lee, Mookkan Prabakaran

**Affiliations:** Temasek Life Sciences Laboratory, 1 Research Link, National University of Singapore, Singapore, Singapore

**Keywords:** Lates calcarifer, immunoglobulin, IGT, betanodavirus, mucosal immunity, tissue expression

## Abstract

Mucosal immunity plays a critical role in the protection of teleost fish against infection, but mucosal immunoglobulin of important aquaculture species unique to Southeast Asia remained greatly understudied. In this study, the sequence of immunoglobulin T (IgT) from Asian sea bass (ASB) is described for the first time. IgT of ASB possesses the characteristic structure of immunoglobulin with a variable heavy chain and four CH4 domains. The CH2-CH4 domains and full-length IgT were expressed and CH2-CH4 specific antibody was validated against full-length IgT expressed in Sf9 III cells. Subsequent use of the anti-CH2-CH4 antibody in immunofluorescence staining confirmed the presence of IgT-positive cells in the ASB gill and intestine. The constitutive expression of ASB IgT was characterized in different tissues and in response to red-spotted grouper nervous necrosis virus (RGNNV) infection. The highest basal expression of secretory IgT (sIgT) was observed in the mucosal and lymphoid tissues such as the gills, intestine and head kidney. Following NNV infection, IgT expression was upregulated in the head kidney and mucosal tissues. Moreover, a significant increase in localized IgT was found in gills and intestines of infected fish on day 14 post-infection. Interestingly, a significant increase in NNV-specific IgT secretion was only observed in the gills of the infected group. Our results suggest that ASB IgT may play an important role in the adaptive mucosal immune responses against viral infection and could potentially be adapted as a tool for the evaluation of prospective mucosal vaccines and adjuvants for the species.

## 1 Introduction

Immunoglobulins (Igs) are glycoproteins produced by the adaptive immune system, serving as the first line of defence against host infection by pathogenic organisms. Until the discovery of immunoglobulin T/Z (IgT/Z) (1, 2), prior knowledge of fish immunoglobulins was restricted to IgM and IgD. The recognition of IgT marked a turning point in the better understanding of fish mucosal immunity and opened up new possibilities for manipulation of the teleost immune system for greater protection against pathogens.

Cartilaginous and bony fishes are widely considered to produce three major classes of immunoglobulin, namely the IgM, IgD and IgT (1, 3). Among the three Igs, IgM is the most prevalent Ig found in the systemic circulation and the main mediator of adaptive B cell responses in fish (4). The function of IgD remains largely unknown, but recent studies have suggested a role of IgD in the maintenance of mucosal homeostasis in the teleost gut and gills and the redemption of auto-reactive B cells (5, 6). IgT is the equivalent of mucosal immunity in teleost and exists as monomers in serum and polymeric forms in mucosal tissues (7). Secretory IgT (sIgT) is produced by a specialized lineage of IgT^+^ B cells primarily found in the mucosal-associated lymphoid tissues (MALTs) (8). Six different MALTS have been identified thus far and are respectively known as nasopharynx-associated lymphoid tissue (NALT) (8, 9), gut-associated lymphoid tissue (GALT) (7), skin-associated lymphoid tissue (SALT) (10), gill-associated lymphoid tissue (GIALT) buccal mucosa-associated lymphoid tissue and pharyngeal mucosa-associated lymphoid tissue (11). IgT found in the MALTs is known to coat mucosal pathogens and microbiota, particularly in the gut and gill to maintain mucosal homeostasis (12). Additionally, IgT^+^ B cells have been observed to be mobilized to the infection site *in-vivo* (13).

Characterization of IgT in rainbow trout (*Oncorhynchus mykiss*), turbot (*Scophthalmus maximus*), Atlantic salmon (*Salmo salar*) and Gilthead Sea bream (*Sparus aurata*) have demonstrated a protective role of IgT against viral, bacterial and parasitic infections (14, 15) through the production of antigen-specific sIgT and IgT^+^ B cells (8). As the first two teleost species from which the IgT was characterized, the IgT gene has been extensively investigated in zebrafish and rainbow trout. However, for others commercially important aquaculture species such as the Asian Seabass (ASB) or barramundi (*Lates calcarifer*), the IgT gene and function have remained largely unknown. ASB is a valuable catadromous aquaculture species belonging to the order Perciformes with wide geographic distribution across Southeast Asia (SEA) ranging from Taiwan to Northern Australia and India (16). Production of ASB in SEA, excluding Cambodia, was estimated to be 73,000 tons, with commercial values of US$ 186 million in 2022 (17). Despite extensive selective breeding for disease-resistance traits, ASB larval and juvenile stages remain highly susceptible to bacterial and viral infectious diseases. In ASB larvae, viral nervous necrosis disease due to betanodavirus or nervous necrosis virus (NNV) is especially virulent and hatchery outbreaks of the disease can result in high mortality and massive economic losses (18). In the current study, the sIgT gene of ASB was identified and sIgT expression profile was analyzed in a series of mucosal and non-mucosal tissues by real-time quantitative PCR for healthy and NNV-infected ASB. The basal expression of IgT was investigated before and after an NNV challenge to determine the dynamics and involvement of ASB IgT. Localized secretion of IgT was also quantitated in the intestines and gills in response to a viral infection. Understanding ASB mucosal immune response upon exposure to infectious agents will improve insight into the function of IgT and its importance in the development of efficacious mucosal vaccines against pathogens.

## 2 Materials and methods

### 2.1 Fish

Healthy Asian Seabass (2 ± 0.5g) were obtained from a commercial hatchery (Allegro Aqua, Singapore) and fish (n=120) were divided equally into four 200 L recirculating tanks each equipped with a standard biofiltration system and aeration at the experimental marine aquarium facilities of Temasek Life Sciences Laboratory (TLL, Singapore). Fish were maintained in 30 ± 1°C UV-irradiated seawater, with a 12:12 light and dark photoperiod cycle. All fish were fed with a commercial pellet diet at 5% body weight per day. During the acclimatization period, 5% of the fish were randomly sampled and analysed for NNV and bacterial diseases common to the species. Experimental animals were ascertained to be good health and tested negative for common seabass bacterial pathogens and betanodavirus prior to start of experiments. Animal experiments for this study were approved by the Institutional Animal Care and Use Committee (IACUC) of TLL (IACUC approval number TLL(F)-22-011).

### 2.2 Cloning of IgT cDNA

Healthy Asian seabass were euthanized with an overdose of Tricaine methane-sulfonate (MS-222) and the head kidney, gills and intestine were harvested and homogenized immediately in TRIzol reagent (Invitrogen, Carlsbad, CA, USA) for RNA extraction. Total RNA was isolated in accordance with the manufacturer’s instructions. The concentrations of RNA were adjusted to 2 µg and reverse transcribed to cDNA using AMV Reverse Transcriptase according to the manufacturer’s instructions (Promega). IgT primers (**Table S1**) were designed based on IgT heavy chain conserved regions obtained through BLAST alignment of IgT amino acid sequences of *Dicentrarchus labrax* (Accession Number KM410929), *Oncorhynchus mykiss* (Accession number AY870263), *Sparus aurata* (Accession Number KX599200), *Siniperca chuatsi* (DQ16660) and *Larimichthys crocea* (Accession Number MW450786) retrieved from NCBI GenBank. The full-length cDNA sequence was amplified and ligated into pJET 1.2 CloneJET PCR cloning kit (Thermo Fisher Scientific, Waltham, MA, USA). Following transformation into competent *Escherichia coli* (*E. coli*) XL1-Blue cells, positive clones were screened by ampicillin selection, colony PCR and sequenced.

### 2.3 Sequence and phylogenetic analysis

The full-length secretory IgT (sIgT) nucleotide sequence was translated using the ExPASy Translate (https://web.expasy.org/translate/) and was confirmed using NCBI (Protein Blast). Signal peptide was identified using Signal P-6.0 (https://services.healthtech.dtu.dk/service.php?SignalP). The variable, diversity and joining (VDJ) segments of sIgT and immunoglobulin domains were predicted with NCBI (IGBLAST) and NCBI Conserved Domain Database (https://www.ncbi.nlm.nih.gov/Structure/cdd/wrpsb.cgi) respectively. The N-glycosylation sites were established with the use of NetNGlyc-1.0 (https://services.healthtech.dtu.dk/service.php?NetNGlyc-1.0). PROMALS3D (http://prodata.swmed.edu/promals3d/promals3d.php) and Clustal Omega (https://www.ebi.ac.uk/Tools/msa/clustalo/) were used for alignment and comparison of Asian seabass sIgT amino acid sequences to other teleost species. A phylogenetic tree with IgT, IgM and IgD sequences from related teleost species was constructed with MEGA X Software using neighbor-joining method. Bootstrap values supporting tree nodes were derived from 1,000 replicates. All positions containing gaps or missing data were eliminated using complete deletion. The theoretical isoelectric point (pI) and molecular weight (MW) of sIgT protein were deduced with the use of Expasy-ProtParam tool (https://web.expasy.org/protparam/).

### 2.4 Cloning, expression and purification of IgT in *E. coli* expression system

The full-length IgT gene was amplified and cloned into a pET32a plasmid using primers IgT SacI F and IgT XhoI R (**Table S1**). The IgT CH2-CH4-pET32a vector was subsequently created using site-specific recombination (IgT CH2-CH4 SacI F and IgT XhoI R) and the full-length IgT gene was replaced in the IgT-pET32a recombinant vector. The full-length and CH2-CH4 IgT recombinant plasmids were then transformed into *Escherichia coli* BL21 (DE3) respectively and positive clones were screened *via* PCR reaction and confirmed with nucleotide sequencing. After confirmation of the sequences, the positive transformant was inoculated into 40 ml of Luria Bertani (LB) medium containing 100 μg/ml ampicillin and grown at 37°C overnight with shaking at 220 rpm. On the following day, the overnight culture was transferred to a 1L flask of LB medium containing ampicillin and incubated at 37°C with constant agitation up to an optical density (OD600). Isopropyl-b-D-thiogalactopyranoside (IPTG) was added to a final concentration of 1mM and protein expression was performed at 25°C for 6 h with constant shaking. Uninduced and induced cell lysates were analyzed on 12% SDS-PAGE and visualized with Coomassie blue staining.

Following induction, the recombinant IgT proteins were purified using a Ni ^2+^ chelating Sepharose column. Purified proteins were analyzed by SDS-PAGE and Western blot and dialyzed against PBS twice at 4°C overnight. The final protein concentration was determined by A_280_ using nanodrop ND-1000 spectrophotometer.

### 2.5 Cloning and expression of IgT in baculovirus-insect cell expression system

The full-length IgT gene of ASB was amplified with primers IgT NotI F and IgT XhoI R (**Table S1**) and inserted into pFastBac HT A transfer vector. The construct was integrated into the baculovirus genome with DH10BAC™ and the Bac-To-Bac system (Invitrogen). The bacmid DNA with IgT gene was purified for transfection into *Spodoptera frugiperda* (Sf9 III) cells (ATCC) for producing recombinant baculovirus (Bac-IgT). The Sf9 III cells were grown at 27°C in serum-free medium SF-900 III (Invitrogen). Procedures for the generation of recombinant baculovirus were carried out according to the manufacturer’s instructions (Invitrogen). Briefly, 1.2 X 10^6^ Sf9 III cells were plated onto 6-well plates for 1 h. After attachment, 4 µg of recombinant bacmid DNA Bac-IgT and 10 µl Cellfectin II (Invitrogen) were diluted with culture medium and used for transfection of Sf9 III cells. Transfection was performed for 5 h at 27°C with the replacement of fresh SF-900 III medium after incubation. Transfected cells were kept at 27°C for 72 h. Following incubation, the supernatant containing recombinant viruses (Bac-IgT) was utilized for infection of fresh Sf9 III cells. For large scale viral production, Sf9 III cells were infected in suspension cultures of 2×10^6^ cells/ml with the collection of supernatant four days post-infection.

### 2.6 Production of anti-IgT polyclonal antibody

Purified recombinant IgT CH2-CH4 was diluted and emulsified in a 1:1 ratio with complete Freund’s adjuvant to a final concentration of 1 mg/ml for subcutaneous immunization of a New Zealand white rabbit. Following the first immunization, two more immunizations were performed every fortnightly using the same formulation but with 1:1 emulsification with Freund’s incomplete adjuvant. The immune serum was collected 10 days after the final immunization for Western blot analysis and storage at -20°C. BALB/c mice were immunized subcutaneously with 1:1 ratio of complete Freund’s adjuvant and 200 µg of IgT CH2-CH4 purified protein. Two weeks after the first immunization, the mice were immunized with 200 µg of IgT CH2-CH4 diluted in PBS and emulsified in a 1:1 ratio with Freund’s incomplete adjuvant. The immunization was repeated for the third time prior to the harvest of serum for western blot and use in immunofluorescence assays.

### 2.7 Reactivity of anti-CH2-CH4 with full-length IgT expressed in baculovirus-insect cell expression by indirect immunofluorescence assay

Sf9 III cells infected with Bac-IgT virus were preserved with 4% paraformaldehyde (PFA) three days post infection. After fixation, the cells were permeabilized with 0.1% Triton X-100. The expressed IgT protein was stained with 1:200 dilution of mouse or rabbit anti-CH2-CH4 IgT antibodies at room temperature for 1 hour. The cells were then incubated with 1:1000 dilution of Alexa fluor 488 labelled goat anti-mouse IgG (Invitrogen, Gaithersburg, USA) or Alexa fluor 488 labelled goat anti-rabbit IgG (Invitrogen, Gaithersburg, USA) and counterstained with DAPI (Invitrogen). The fluorescence signal was detected with an inverted fluorescence microscope (Olympus, UK) and the images were captured by a digital imaging system (Nikon, Japan).

### 2.8 Quantitative analysis of IgT expression in healthy ASB by real-time PCR

Mucosal and non-mucosal tissues were harvested from six healthy ASB approximately 100 g in weight and individually homogenised in TRIzol reagent (Invitrogen, CarIsbad, CA, USA) for total RNA extraction in accordance with manufacturer’s instructions. The concentrations of RNA were adjusted to 2 µg and reverse transcribed to cDNA using AMV Reverse Transcriptase according to the the manufacturer’s instructions (Promega). Real-time quantitative PCR (qPCR) was carried out with the Quant Studio 5 Real-Time PCR System (Applied Biosystems), using a 384-well layout designed for simultaneously profiling of IgT and housekeeping genes for all individuals of each tissue. Briefly, the cDNA was amplified in 20 µl reaction containing 0.5 µM of forward primer (5’- TCCAGACGAGGACATTGATAAAG -3’), 0.5 µl of cDNA, 0.5 µM of reverse primer (5’- ATGTGCTTCTCGTTCCAGTT -3’) and Go-Taq qPCR Master mix (Promega, Madison, WI, USA). The PCR reaction was performed with the following conditions: 1 cycle of 95°C for 2 min, followed by 40 cycles of 95°C for 15 s and an annealing and extension step at 54°C for 60 s. The IgT expression levels in different fish tissues were normalised against the *β*-actin. IgT expression in tissues was analysed using the 2^- ΔΔCT^ method. All experiments were conducted in triplicate.

### 2.9 Immunohistochemistry

For immunohistochemistry (IHC), gills and posterior intestines from ASB were harvested and fixed in 4% PFA overnight at room temperature. Following fixation, the tissues were gradually dehydrated and embedded in paraffin before being sectioned into 7 μm slices. The paraffin-embedded slices were deparaffinized and hydrated with Histoclear (Thermo-scientific) and ethanol. Antigen retrieval was performed by heating the slides in sodium citrate buffer (10mM sodium citrate, 0.05% Tween 20, pH 6.0) under high pressure for 10 min. The slides were then blocked in 3% skim milk for 1 h at room temperature, followed by incubation with mouse anti-ASB IgT (1:20 dilution) overnight at 4 °C. The following day, the slides were rinsed in PBS thrice and incubated with the FITC labelled rabbit anti-mouse IgG (Dako) at 1:50 dilution for 2 h at room temperature. The slides were then washed thrice in PBS before the nuclei were stained with propidium iodide (PI) for 10 min at room temperature. A final wash was performed with PBS before the slides were mounted with ProLong™ anti-fade mounting reagent (Invitrogen). Microscopy visualization and images were collected using Olympus FV3000 confocal microscope.

### 2.10 IgT expression and secretion in ASB after viral challenge

Healthy Asian seabass juveniles (6-8 g) were divided into two groups (n=20/group) for the nodavirus challenge. Each group of fish were kept in 200L of 30 ± 1°C UV-irradiated seawater recirculation tanks equipped with a standard biofiltration system and aeration. Experimental fish were exposed to a 12:12 light and dark photoperiod cycle throughout the challenge. The challenge group was immersed in PBS containing 10^7^ TCID_50_/ml of RGNNV (19) for 15 minutes. The control group was immersed in PBS alone. Control and challenged animals were sacrificed (n=6/group) at 24 h and 48 h post-treatment and the spleen, gill, skin, head kidney and intestine tissue of individual fish were collected for RNA extraction. As described above, the expression profiles of ASB IgT mRNA in different tissues were amplified with IgT primers and normalized against 18S RNA gene. IgT fold change was quantitated using the 2^- ΔΔCT^ method. All experiments were conducted in triplicate. On day 14, gill and intestine tissues of infected and control fish were harvested for determination of total IgT and NNV-specific IgT and IgM. The intestine was opened longitudinally and rinsed with PBS three times to remove the faeces. Gill arches were excised and rinsed with PBS three times. The gill and intestine were resuspended in 200 ul of PBS and subsequently lysed using a tissue homogenizer. Tissue lysates were spun down at 10000 rpm for 10 min before the collection of supernatants for IgT ELISA.

### 2.11 Measurement of total IgT and anti-NNV-specific IgT or IgM antibodies in gills and intestine by ELISA

Antigen capture ELISA was used to determine total IgT in tissue lysate. Briefly, microtiter well ELISA plates were coated with 1:100 dilution of mouse anti-CH2-CH4 antibody in coating buffer (0.1mol/liter carbonate-bicarbonate, pH 9.6) and incubated at 4°C overnight. After incubation, plates were washed three times and blocked with 3% BSA (Sigma) for 1 h at 37°C. All washes were performed with PBS containing 1% tween-20 (PBS-T). After washing, 100 µl of 1:20 dilutions of gill or intestine mucosal wash samples were added and plates were incubated for 1 h at 37°C. Plates were then washed three times and a 100 µl aliquot containing 1:200 dilutions of rabbit anti-CH2-CH4 IgT was added and incubated for 1 h at 37°C. After three washes, 100 µl of goat anti-rabbit HRP-conjugated antibody (DAKO Cytomation, Copenhagen, Denmark) diluted 1:1000 was added. After the final wash, the color development was visualized by adding 100 µl 3,3’,5,5’-tetramethylbenzidine (Sigma). The absorbance was measured at 450 nm using a microwell plate reader.

Similarly, the NNV-specific IgT or IgM level was determined by indirect ELISA. Purified RGNNV viral antigen was coated onto microtiter well ELISA plates and incubated overnight at 4°C. After washing and blocking with 3% BSA, 100 µl of gill or intestine mucosal wash samples were added and plates were incubated for 1 h at 37°C. Plates were washed three times and a 100 µl aliquot containing 1:200 dilutions of rabbit anti-CH2-CH4 IgT or 1:33 dilutions of anti-ASB IgM monoclonal antibody (Aquatic Diagnostics Ltd., Stirling, UK) was added and incubated for 1 h at 37°C. The color development was then visualized by adding goat anti-rabbit HRP-conjugated antibody or rabbit anti-mouse IgG conjugated to HRP (Dako Cytomation, Denmark) to the respective wells and followed by the addition of 3,3’,5,5’-tetramethylbenzidine (Sigma). The absorbance was measured at 450 nm using a microwell plate reader.

### 2.12 Statistical analysis

All data were expressed as mean ± standard error (SE). The unpaired two-tailed Student’s t-test was performed to determine the significant (P < 0.05) differences between the means of the two groups.

## 3 Results

### 3.1 Identification and characterization of Asian seabass IgT

**Figure 1:**
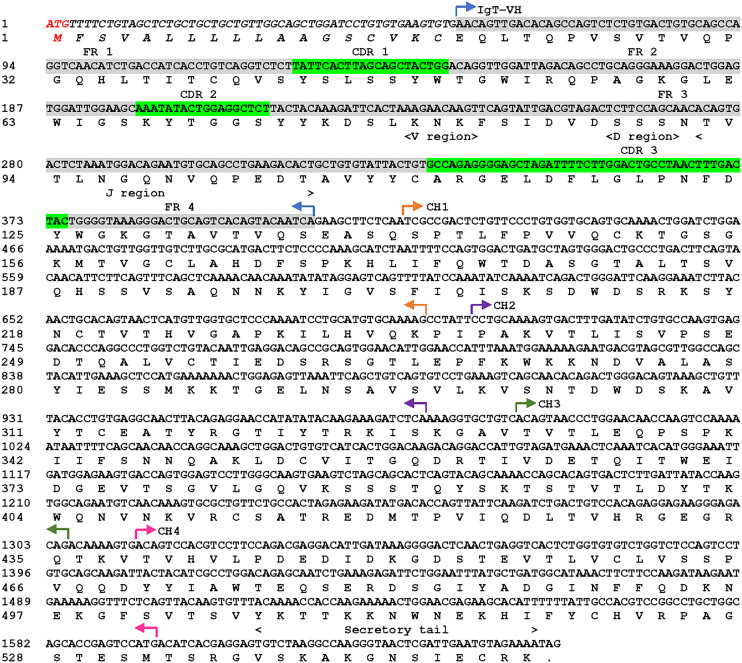
Asian seabass (*L. calcarifer*) secreted form of IgT nucleotide and amino acid sequence (sIgT, GenBank accession number OQ108524). The VH, predicted VDJ segment, four CH regions (CH1, CH2, CH3 and CH4) and C-terminal secretory tail are depicted in the sequence. The predicted signal sequence of ASB sIgT heavy chain is in italics, framework region (FR) and complementarity determining region (CDR) were highlighted in grey and green respectively.

The cloned cDNA sequence of sIgT (GeneBank accession number: OQ108524) from Asian seabass was obtained by amplification of two fragments using two primer pairs (**Table S1**). The open reading frame (ORF) of ASB sIgT was found to be 1650 bp long and encodes for a protein of 550 amino acid (aa) residues, among which are a putative 18-aa signal peptide, a 114-aa variable region, 93-aa CH1, 91-aa CH2, 104-aa CH3 and 94-aa CH4 (**Figure 1**). The full-length ASB secretory IgT was predicted to be 60.47 kDa in molecular weight with a theoretical isoelectric point of 7.82 and contains a potential N-glycosylation region (**Figure 1**).

### 3.2 Multiple sequence alignment and phylogenetic analysis

**Figure 2:**
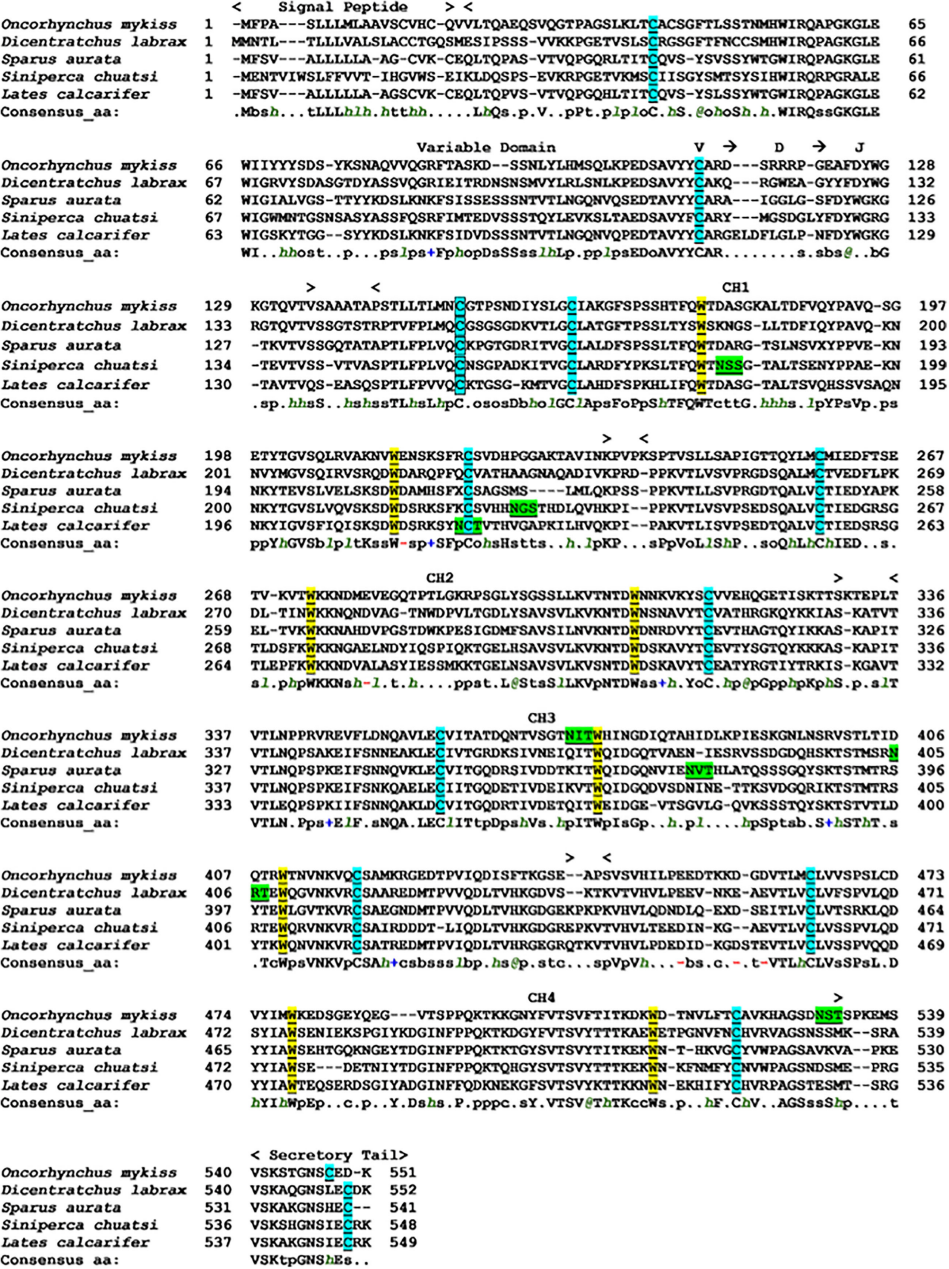
Alignment of Asian seabass secretory IgT heavy chain amino acid sequence with other known secretory IgT heavy chain fish species performed with PROMALS3D. The sequence was divided into predicted signal sequence, variable domain, predicted VDJ segment, four CH regions (CH1, CH2, CH3 and CH4) and C-terminal secretory tail are depicted above the sequences. The conserved cysteines residues that are underlined and highlighted in light blue are expected to form the intra-chain disulfide bonds, while the cysteine residues in the black square box are expected to constitute the disulfide bond to link the light chains to form the complete Ig molecule. The tryptophan residues were underlined and highlighted in yellow. Underlined and highlighted in green are the predicted N-glycosylation site. Consensus amino acid symbols are: conserved amino acids are in bold and uppercase letters; aliphatic (I, V, L): *l*; aromatic (Y, H, W, F): *@*; hydrophobic (W, F, Y, M, L, I, V, A, C, T, H): *h*; alcohol (S, T): o; polar residues (D, E, H, K, N, Q, R, S, T): p; tiny (A, G, C, S): t; small (A, G, C, S, V, N, D, T, P): s; bulky residues (E, F, I, K, L, M, Q, R, W, Y): b; positively charged (K, R, H): **+**; negatively charged (D, E): -; charged (D, E, K, R, H): c.

The multiple alignments of amino acid sequences of ASB sIgT and IgT heavy chain sequences from four teleost species were performed using PROMALS3D. The result indicated a conservation of ASB sIgT for the prototypical Ig structure, with the presence of a signal peptide region, variable domain, VDJ segment and four CH domains followed by a secretory tail (**Figure 2**). Homology comparison of ASB sIgT against other related teleost species revealed a 62.03% sequence identity to sIgT of *Sparus aurata* (**Table S2**). The phylogenetic analysis found a clear segregation between the IgT, IgM and IgD sequences of all species, with the identified sequence from ASB classified under IgT and clustered accordingly with similar isotypes of other species (**Supplementary Figure 1**).

### 3.3 Expression of recombinant full-length and CH2-CH4 IgT in *E. coli* BL21 (DE3)

**Figure 3:**
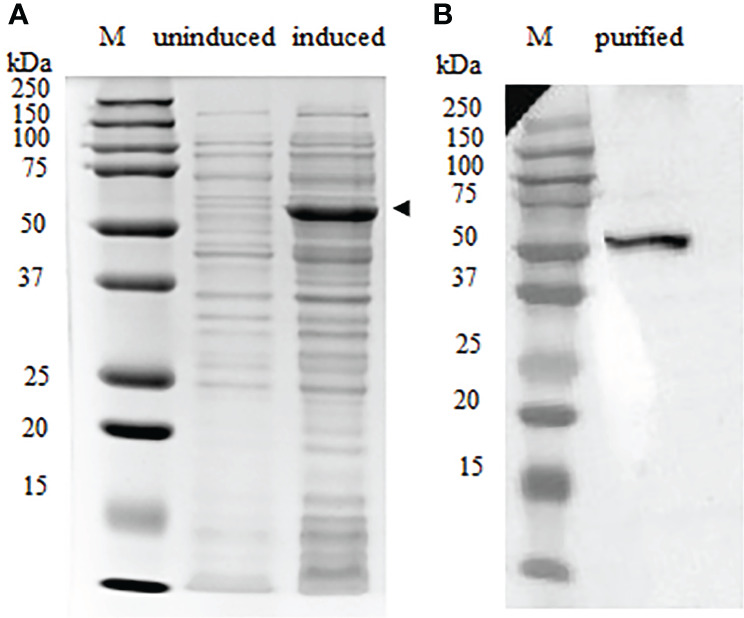
Expression of CH2-CH4 rIgT from *E. coli* BL21 (DE3) under reducing conditions. **(A)** Comparison of CH2-CH4 rIgT expression (black arrow) in uninduced and induced *E. coli* BL21 (DE3) lysates. **(B)** Purified CH2-CH4 rIgT. M: marker.

The full-length recombinant IgT (rIgT) and CH2-CH4 domains of IgT were subcloned into pET32a expression plasmid and transformed into *E. coli BL21* (DE3) respectively. Following IPTG induction, BL21 *E. coli* transformed with the full-length IgT recombinant plasmid can be observed to express a band of approximately 78 kDa on SDS-PAGE (**Supplementary Figure 2**). The band size was consistent with the predicted size of full-length ASB IgT (~60 kDa) fused to a thioredoxin-hist tag (~18 kDa) under reducing conditions. CH2-CH4 IgT expressed in *E. coli* BL21 (DE3) was observed to produce a band of approximately 52 kDa (**Figure 3A**), corresponding to the predicted size of CH2-CH4 rIgT (~34 kDa) and thioredoxin-his tag (~18 kDa) under reducing conditions. Following cell lysis, the recombinant CH2-CH4 rIgT was purified using nickel affinity chromatography (**Figure 3B**) for immunization in rabbits and mice to produce anti-ASB CH2-CH4 IgT polyclonal antibody.

### 3.4 Reactivity of anti-CH2-CH4 antibody with full-length IgT expressed in Sf9 III cells

**Figure 4:**
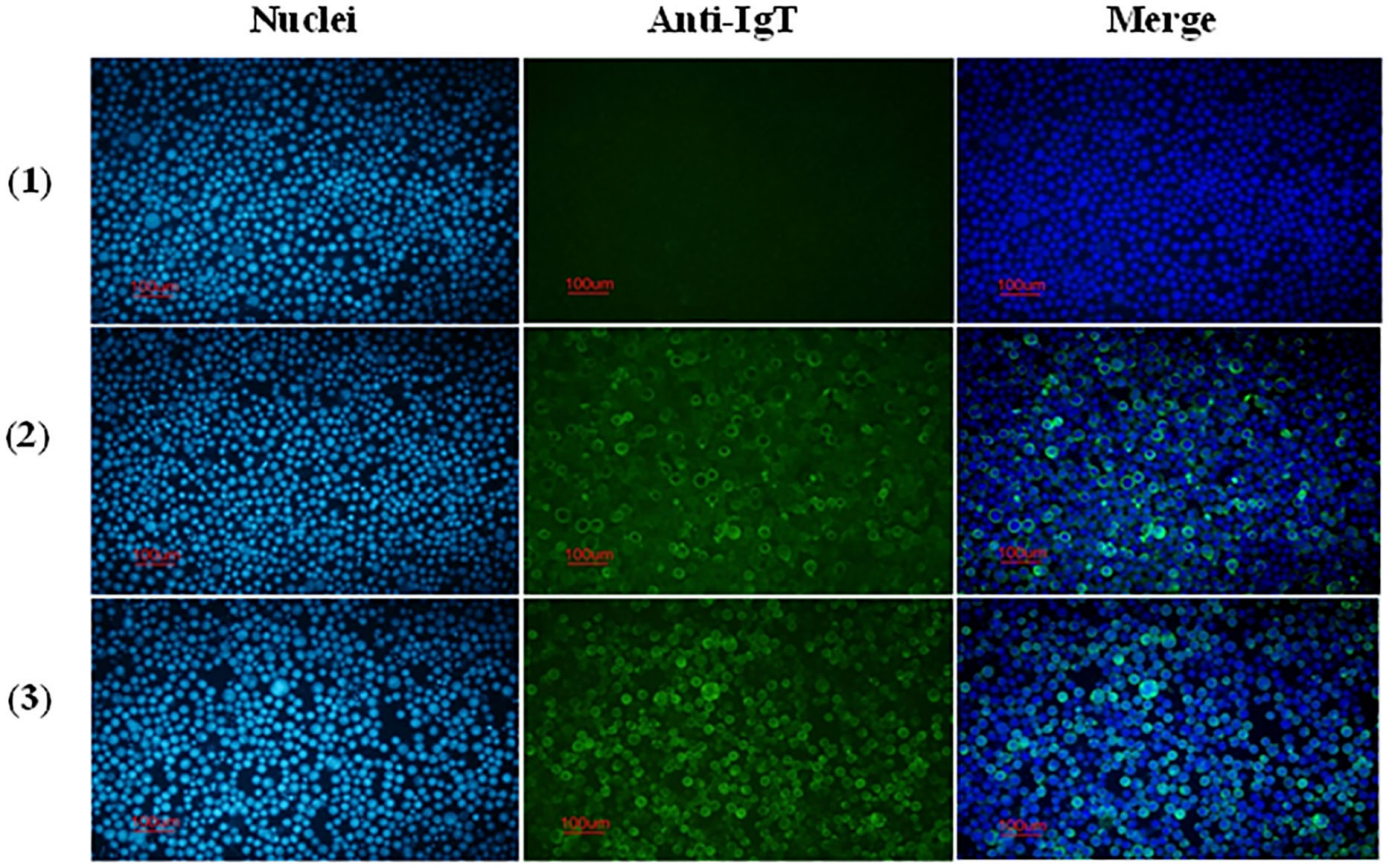
Reactivity of anti-CH2-CH4 IgT antibodies with recombinant full-length IgT expressed in Sf9 III cells. Indirect immunofluorescence staining on control and Bac-IgT infected Sf9 III cells using mouse and rabbit anti-CH2-CH4 rIgT-specific antibodies. Control Sf9 III (1) and Bac-IgT infected Sf9 III (2) cells stained with mouse anti-CH2-CH4 IgT antibody (green) and DAPI (blue). IgT-transfected Sf9 III cells (3) stained with rabbit anti-CH2-CH4 IgT antibody (green) and DAPI (blue).

The reactivity of anti-CH2-CH4 IgT antibodies against full-length Bac-IgT infected Sf9 III was confirmed by indirect immunofluorescence assay. As shown in **Figure 4**, mouse and rabbit anti-CH2-CH4 IgT antibodies were capable of recognizing full-length IgT expressed in Bac-IgT-infected Sf9 III cells. In order to assess if Sf9 III cells were uniquely expressing the ASB rIgT, full-length IgT expressed by Sf9 III cells was probed with anti-ASB IgM antibody. Immunofluorescence staining revealed a lack of cross-reactivity between ASB IgM and ASB rIgT expressed in Sf9 III cells (data not shown).

### 3.5 Immunofluorescence analysis of IgT-positive cells and IgT expression in gill and intestine tissue of ASB

**Figure 5:**
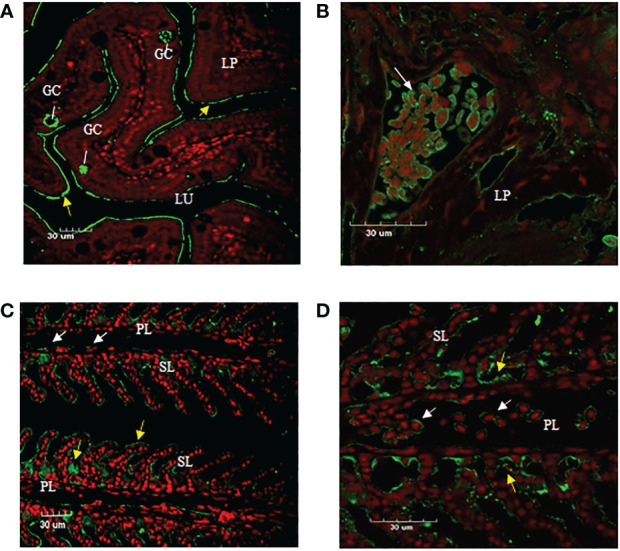
Immunofluorescence staining of IgT-expressing cells in gill and intestine of ASB. Gill and intestinal tissue samples were embedded in paraffin and sectioned. Immunohistochemical staining was carried out using mouse anti-CH2-CH4 IgT antibody followed by rabbit anti-mouse FITC. **(A)** Presence of goblet cells (GC) secreting mucus in the lamina propria (LP) and plausibly secreted IgT (yellow arrows) lining the mucosal surface of the intestine. **(B)** Enlarged image of IgT+ cells presented in the lamina propria (LP) of the intestine (white arrow). **(C)** Localization of IgT+ cells (white arrows) in the primary lamellae (PL) and possibly secreted IgT (yellow arrows) coating the surface of secondary lamellae (SL) in the gill of ASB. **(D)** Magnified image of IgT+ cells located in primary lamellae of ASB gill (white arrows). ASB intestine and gill were stained with mouse anti-IgT (green) and propidium iodide (red). Images are representative of at least three independent experiments.

Immunofluorescence staining of ASB gill and intestine tissue section using anti-CH2-CH4 IgT antibody was undertaken to examine the distribution of IgT expression and IgT positive cells in the two mucosal tissues. The results suggested that the mouse anti-CH2-CH4 IgT antibody could recognize IgT-positive cells in the lamina propria of the ASB intestine (white arrows) (**Figure 5B**). In the gills, IgT expression can be observed on individual cells in the primary lamellae (white arrows) (**Figures 5C, D**). IgT expression was also detected in the external mucous layer (yellow arrows) lining the gill (**Figures 5C, D**) and intestine (**Figure 5A**) and within goblet cells (**Figure 5A**).

### 3.6 Basal expression of IgT in ASB tissues

**Figure 6:**
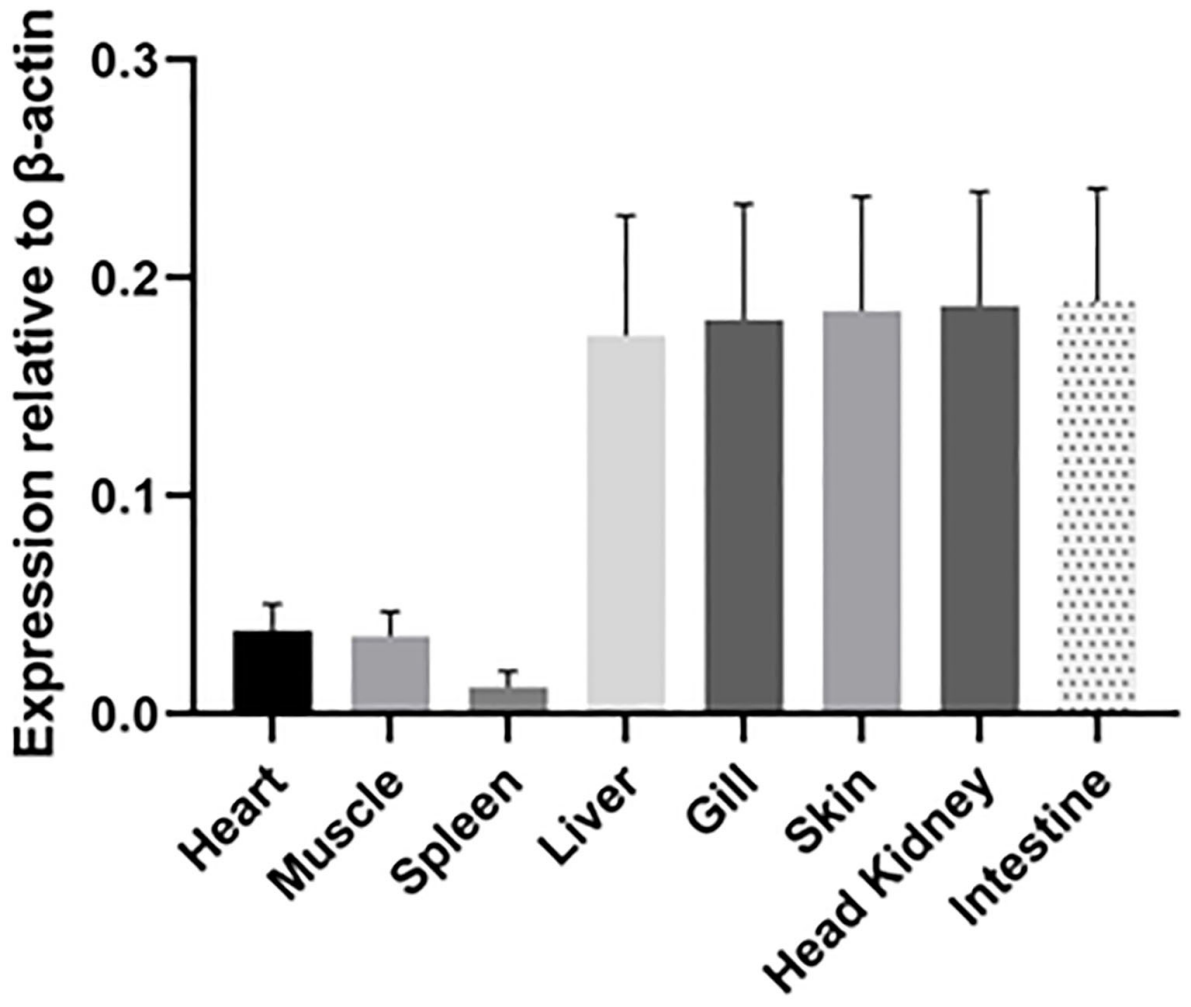
Relative expression of IgT in ASB tissues. Basal IgT mRNA expression in eight ASB tissues (heart, muscle, spleen, liver, gill, skin, head kidney and intestine). Total RNA from various tissues of six ASB was individually isolated and transcribed into cDNA. IgT expression was detected using real-time PCR and ASB IgT-specific primers. The IgT mRNA expression level was normalized to transcripts of *β*-actin and expressed as the mean ± SE.

IgT was expressed in all tested tissues (heart, muscle, spleen, liver, gill, skin, head kidney and intestine) of ASB (**Figure 6**). High IgT expression levels were found in the liver, gill, skin, head kidney and intestine, while low IgT transcription levels were observed in the spleen. IgT expression profiles in tested tissues indicated that ASB IgT transcripts were constitutively expressed with the highest expression levels associated with lymphoid and mucosal tissues such as the head kidney, skin, gill and intestine.

### 3.7 Modulation of IgT expression and secretion after NNV infection

**Figure 7:**
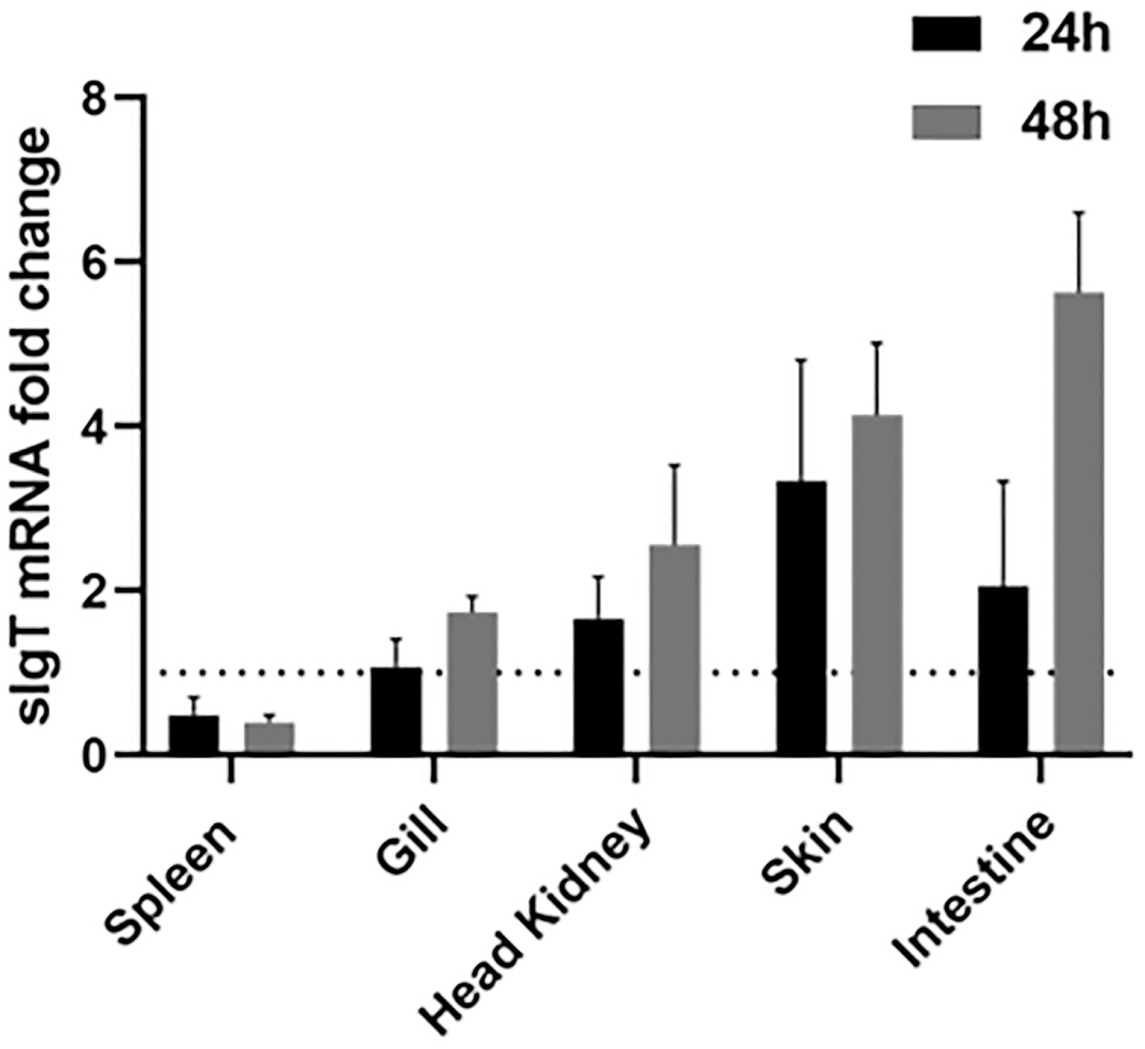
IgT expression in ASB mucosal tissues after exposure to NNV. Modulation of IgT mRNA levels in five tissues of six ASB (spleen, gill, skin, head kidney and intestine) 24 h and 48 h after RGNNV immersion. Total RNA from various tissues was isolated and transcribed into cDNA followed by detection of IgT expression using real-time PCR. IgT gene expression was calculated using the *18S RNA* as a reference gene and the fold change in IgT mRNA expression level was normalized by comparison to the control group. Data were expressed as the mean ± SE. Dotted line represents the baseline of non-challenged fish.

Expression of IgT was analyzed in the lymphohematopoietic organs (head kidney and spleen), gill, intestine and skin. Infection by RGNNV was found to stimulate an increase in IgT expression in the gill, head kidney, skin and intestine when compared to the control (**Figure 7**). The gills of challenged ASB exhibited a two-fold increase in IgT expression at 48 h post-infection compared to the control animals. In the head kidney, IgT expression was upregulated two-fold at 24 h post-challenge with an elevation of close to three-fold 48 h after infection. The strongest response of sIgT was observed in the skin and intestine. IgT transcription in the skin increased by close to four-fold 24 h and 48 h after the challenge. In contrast, a two-fold increase was observed in the intestine of infected fish at 24 h post-infection with an increment to 6-fold at 48 h post-exposure.

**Figure 8:**
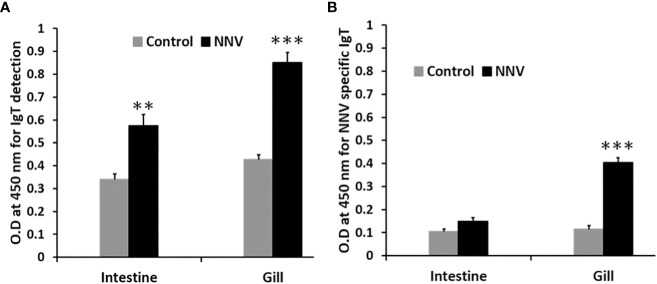
Comparison of total IgT and NNV-specific IgT levels in the gill and intestine of control and ASB infected with RGNNV. **(A)** Total mucosal IgT antibody level was determined by antigen capture ELISA. **(B)** NNV-specific IgT antibody level was determined by indirect ELISA. Infection was performed by immersion of ASB with 10^5^ TCID50 of RGNNV, the organs were collected 14 days post-infection for analysis. Data is a representative of the mean ± SE of 6 fish (**, P<0.01; ***, P<0.001).

ASB exposed to RGNNV through immersion was found to secrete significantly higher levels of mucosal IgT in the intestine (P<0.01) and gills (P<0.001) 14 days post-challenge in contrast to control fish (**Figure 8A**). Additionally, a significant increase in NNV-specific IgT levels (P<0.001) was found in the gills of infected fish as compared to the control group (**Figure 8B**). In contrast, no significant difference in NNV-specific IgM levels was observed in the gills and intestines of infected and control ASB on day 14 post-infection (data not shown).

## 4 Discussion

Antigen recognition, opsonization and neutralization of pathogens are crucial functions provided by mucosal immunoglobulins (Igs) for the protection of teleost against infections. As the dominant Ig in teleost mucus, IgT is critical for the maintenance of mucosal health and homeostasis. Current knowledge on IgT has predominantly been derived from teleost species such as zebrafish and salmonids and only recently has the topic been expanded to include teleost species of commercial interest. Thus, the IgT gene, function and role in response to pathogens and vaccination strategies for many economically important aquaculture species in Asia remain understudied. The current study presents the first description of IgT from ASB and characterizes its response to an NNV infection.

Sequence analysis revealed conservation of ASB sIgT for the prototypical characteristic of immunoglobulin. The identified ASB sIgT sequence contains a V_H_ region, four Cτ domains (CH1-CH4), a secretory tail and conserved cysteine and tryptophan residues necessary for the formation of intra/interchain disulfide bonds and proper immunoglobulin architecture. Characterization of IgT heavy chain in most teleost have found an arrangement of four CH (CH1, CH2, CH3, CH4) domains apart from species such as the fugu and stickleback that possesses lesser CH numbers in non-consecutive order (20, 21). In the case of the ASB, the heavy chain of IgT was shown to retain the prototypical arrangement and number observed previously in perch-like fish (14, 22, 23). Additionally, amino acid sequence comparisons of ASB sIgT revealed the highest conservation to sIgT of the gilthead sea bream (GSB) (*Sparus aurata*), a fellow member of the Perciformes order. In both species, sIgT expression in the head kidney was observed to increase 48h after NNV challenge despite the difference in route of infection (14).

Full-length and CH2-CH4 IgT recombinant products were consistent with their predicted molecular mass based on the amino acid sequences and published literature on recombinant teleost IgT expressed in a prokaryotic system (24, 25). Among the two IgT proteins, recombinant CH2-CH4 IgT was found to be more soluble and better suited for the generation of anti-ASB IgT polyclonal antibodies. Anti-CH2-CH4 IgT antibody was observed to recognize full-length IgT expressed in the baculovirus-insect cell expression system.

Detection of IgT expression in teleost mucosal tissues has previously been established through either immunofluorescence staining or *in-situ* hybridization (7, 22, 24, 26, 27). Previous use of immunofluorescence staining has identified IgT^+^ B cells in the epithelium of gill filaments and within the intestinal epithelium and lamina propria of the intestine in multiple teleost species (22, 24, 26). In this study, IgT-expressing cells were similarly located in the lamina propria of the intestine and primary lamellae (PL) of the gills of the ASB. The identification of IgT^+^ cells in the gill and intestine tissue of ASB represents the first instance whereby IgT-expressing cells are visualized in ASB tissues. As mucosal tissues exposed to the environment, the teleost gut and gills serve as physical barriers against infection by pathogens and possess the ability to respond to pathogenic challenges and microbial colonization by the production of specialized IgT^+^ B cells and pathogen-specific IgT (7, 27).

As with the role of mucosal immunoglobulin, basal expression of sIgT was found to be the highest in mucosal-associated lymphoid tissues (MALTs) such as the intestine, gill and skin. Expression of IgT in the lymphohematopoietic organs revealed low levels of IgT transcripts in the spleen, while the head kidney showed high IgT transcription levels. Analysis of sIgT expression in tissues of GSB and European sea bass has likewise found a trend for higher IgT expression in the gill, intestine and head kidney and lower IgT transcription in the spleen (14, 22). In the Nile tilapia, the highest IgT expression was found in the spleen (23), while flounder and turbot exhibited high IgT expression in the gill, spleen, liver, head kidney and trunk kidney (15, 28). Taken together, the results suggested a tissue specificity for basal sIgT expression with variation in expression levels between teleost species. Exposure of teleost to infectious agents or immunizing antigen was demonstrated to modulate IgT expression through stimulation of the adaptive mucosal immunity (14, 22, 23). European sea bass infected with nodavirus exhibited increased IgT transcription in the gill and spleen post-infection (22). In contrast, immersion vaccination of largemouth bass induced significantly higher IgT mRNA levels in mucosal tissues such as the gill and intestine (23). In the current study, RGNNV immersion was found to elicit a higher response of IgT expression in mucosal tissues of ASB with the strongest response observed in the MALTs ie., gill, skin and intestine. Following NNV infection, the increase in IgT transcription in the mucosal tissues was simultaneously supported by a significant increase in total IgT. However, only a significant increase in NNV-specific IgT levels was detected in the gills of infected fish. The higher levels of NNV-specific IgT detected in the gills when compared to the intestine of infected fish were consistent with the route of infection. In a water-borne challenge, the teleost gills constitute the primary point of entry and replication for invasive pathogens. A study of immersion NNV challenge and immune responses of seven-band grouper has found early virus replication in the gills of infected fish prior to dissemination to the brain (29). Stimulation of MALTs through immersion challenge was found to mostly enhance localized production of antigen-specific mucosal IgT with rare induction of systemic IgT (14, 15, 23, 30).

In the present study, the secreted form of IgT was cloned and characterized for the first time from *L. calcarifer*. Furthermore, immunofluorescence staining provided a first look at the IgT expression patterns and presence of IgT+ cells in the gill and intestine of the healthy ASB. The involvement of ASB IgT in the adaptive mucosal immune response and induction of antigen-specific IgT through mucosal exposure suggest a protective role of ASB IgT in the mucosal immune response against viral pathogens. Our results provided a strong basis for further studies on IgT involvement in viral infection to elucidate new information to understand the specific contributions of IgT against infections in the Asian seabass. The inclusion of ASB IgT response for evaluating novel vaccine designs and mucosal adjuvants will aid in developing effective mucosal vaccines to combat infectious diseases in ASB aquaculture.

## Data availability statement

The datasets presented in this study can be found in online repositories. The names of the repository/repositories and accession number(s) can be found in the article/**Supplementary Material**.

## Author contributions

Conceived and designed the experiments: MP, JC and LC. Performed the experiments: JC, LC and SL. Analyzed the data: MP, JC and LC. Contributed materials/analysis tools: LC. Writing – original draft: JC. Writing-review and editing: MP, JC and LC. All authors contributed to the article and approved the submitted version.
