# Supplementary material for: Identification and characterization of immunoglobulin tau (IgT) in Asian Seabass (Lates calcarifer) and mucosal immune response to nervous necrosis virus
Source: Front Immunol. 2023 Feb 20;14:1146387. doi: 10.3389/fimmu.2023.1146387 (PMC9986254; doi:10.3389/fimmu.2023.1146387)
Supplement: Supplementary file 1 [file DataSheet_1.docx]

Supplementary Material

Identification and characterization of Immunoglobulin Tau (IgT) in Asian Seabass (*Lates calcarifer)* and mucosal immune response to Nervous necrosis virus

**Table S1. Primers used for gene cloning and qPCR in this study**

| **Primer** | **Oligonucleotide Sequence (5’- 3’)** | **Application** | **Accession number** |
| --- | --- | --- | --- |
| ASB-IgT-A F | ATGTTTTCTGTAGCTCTGCTGCTGCTG | IgT identification from ASB genome |  |
| ASB-IgT-A R | GACCAGACACACCAGAGTGAC |  |  |
| ASB-IgT-B F | ATGACTCCAGTTGTTCAAGATCTGACTGTCCACA |  |  |
| ASB-IgT-B R | CTATTTTCTACATTCAATCGAGTTACCCTTGGC |  |  |
| IgT NotI F | CAACTAGTGCGGCCGCATGTTTTCTGTAGCT | IgT protein expression in baculovirus expression system |  |
| IgT XhoI R | CCGCTCGAGTTTTCTACATTCAATCGAGTT | IgT protein expression in *E. coli* and baculovirus expression systems |  |
| IgT SacI F | **C**GAGCTCATGTTTTCTGTAGCTCTGCTGC | IgT protein expression in *E. coli* |  |
| IgT CH2-CH4 SacI F | **C**GAGCTCCCCAAAATCCTGCATGTGCAAAAGC | IgT CH2-CH4 protein expression in *E. coli* |  |
|  |  |  |  |
| IgT-qPCR F | TCCAGACGAGGACATTGATAAAG | qRT-PCR |  |
| IgT-qPCR R | ATGTGCTTCTCGTTCCAGTT |  |  |
| *β*-actin F | TACCACCGGTATCGTCATGGA |  | GU188683.1 |
| *β*-actin R | CCACGCTCTGTCAGGATCTTC |  |  |
| 18S-rRNA F | AACGAGACTCCGGCATGCTA |  | GQ507431.1 |
| 18S-rRNA R | CCGGACATCTAAGGGCATCA |  |  |

**Table S2. Protein sequences of IgT heavy chain used for phylogenetic tree analysis and multiple sequence alignment.**

| **Species** | **IgT heavy chain (% identity)** | **NCBI Accession Number** |
| --- | --- | --- |
|  | Signal Sequence to Stop Codon |  |
| *Lates calcarifer* | 100 | OQ108524 |
| *Sparus aurata* | 62.03 | KX599200 |
| *Siniperca chuatsi* | 59.78 | DQ016660 |
| *Scophthalmus maximus* | 56.58 | KU934278 |
| *Paralichthys olivaceus* | 55.72 | KX174302 |
| *Dicentrarchus labrax* | 52.35 | KM410929 |
| *Epinephelus coioides* | 50.65 | MN782319 |
| *Larimichthys crocea* | 50.54 | MW450786 |
| *Oncorhynchus mykiss* | 42.43 | AY870263 |
| *Plecoglossus altivelis* | 32.80 | AB921958 |
| *Oreochromis niloticus* | 28.94 | KP685367 |

**Supplementary Figure 1.** Phylogenetic tree analysis of immunoglobulin family members from Asian seabass and other fish species. Asian seabass IgT was marked with a blue square. The tree was constructed by neighbor-joining method using Mega X software and the node values represented the percent of bootstrap confidence derived from 1000 replicates. The GenBank accession number for each sequence followed the common species name.


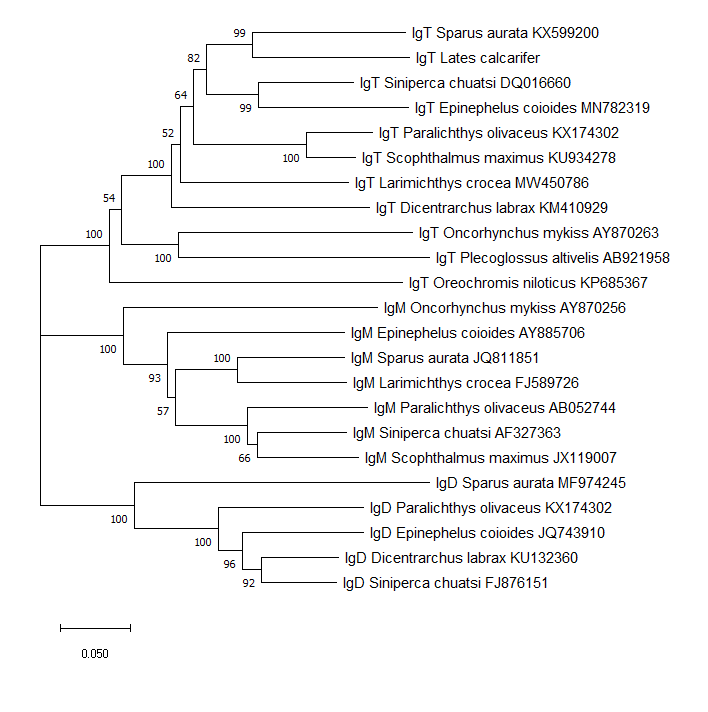


IgT

IgM

IgD


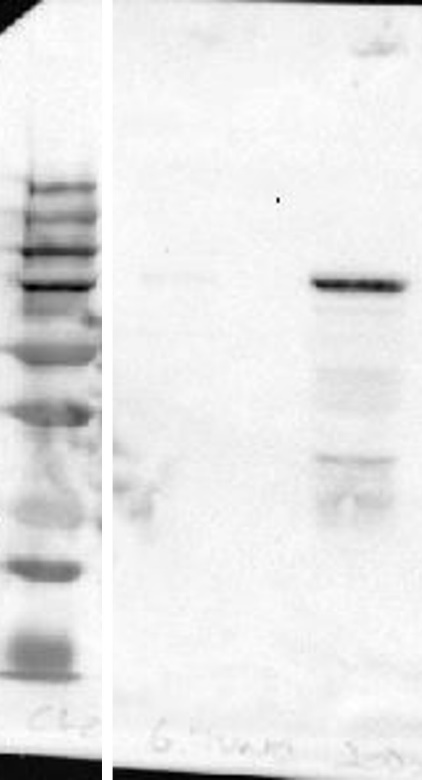


250

kDa

75

50

25

150

100

37

20

M


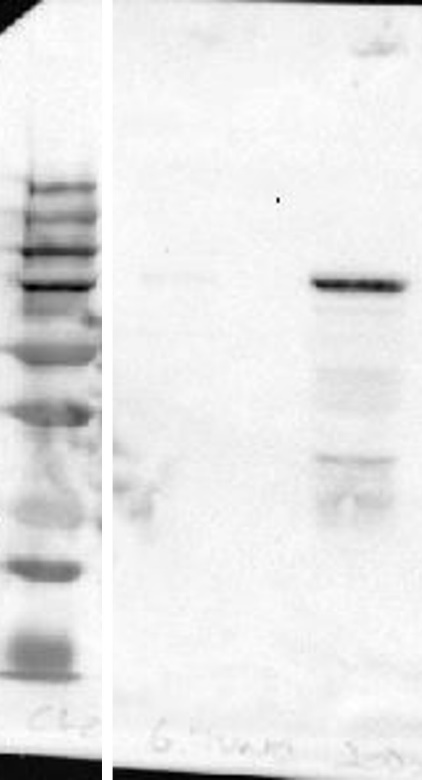


**A**

**Supplementary Figure 2.** Expression of full-length ASB rIgT from *E.coli* BL21 (DE3) under reducing conditions. Western blot analysis of full-length rIgT induced *E. coli* BL21 (DE3) lysates using mouse anti-hist tag antibody M: marker.
